# Are *Salmonella*-Induced Gastroenteritis Neglected in Developing Countries? Feedback from Microbiological Investigations in N’Djamena Hospitals, Chad

**DOI:** 10.1371/journal.pone.0136153

**Published:** 2015-08-27

**Authors:** Djim-adjim Tabo, Sophie A. Granier, Colette D. Diguimbaye, Muriel Marault, Anne Brisabois, Baïzina Mama, Yves Millemann

**Affiliations:** 1 Faculté des Sciences Exactes et Appliquées (FSEA), Université de N’Djamena, N’Djamena, Chad; 2 Université Paris-Est, ENVA, Unité MASQ (Microbiologie des Aliments, Sécurité et Qualité), Maisons Alfort, France; 3 Université Paris-Est, ANSES, Laboratoire de Sécurité des Aliments, Mission Antibiorésistance, Maisons-Alfort, France; 4 Laboratoire de Recherches Vétérinaires et Zootechniques de Farcha (LRVZ/ F), N’Djamena, Chad; Ross University School of Veterinary Medicine, SAINT KITTS AND NEVIS

## Abstract

*Salmonella* is considered to be one of the main pathogens causing human gastroenteritis worldwide. Looking for *Salmonella* in Africa in patients suffering from gastroenteritis is rather unusual, and the use of antibiotics is not subject to any regulation. This study intends for stressing the possible prominent importance of *Salmonella* in digestive diseases in Africa as well as identifying antimicrobial resistance of *Salmonella* isolates from faeces samples of human origin. All samples were collected from five N’Djamena hospitals, from patients suffering from diarrhoea. The collecting was undertaken over two periods of six months each: from August 2010 to January 2011 and from September 2011 to February 2012. *Salmonella* isolates were obtained by standard cultivation and serotyping methods. A total of 43 *Salmonella* isolates were identified, belonging to 21 different serovars. The most prevalent serovar was *Salmonella* Stanleyville (n = 7), followed by *S*. Anatum (n = 4) and *S*. Kottbus (n = 3). The other serovars were under-represented. The majority of these isolates were susceptible to all antibiotics tested (CLSI Standards), except two *S*. Enteritidis isolates that exhibited resistance to fluoroquinolones. The different serovars and antibiotic resistance profiles that were observed highlight the substantial diversity of *Salmonella* in N’Djamena, Chad. Roughly, one out of ten patients who consulted for gastroenteritis was shedding *Salmonella* spp. and none of them would have been diagnosed outside the context of this research program. This study may encourage local clinicians to explore more often salmonellosis suspicion in their daily practice.

## Introduction

*Salmonella* is an important food-borne pathogen responsible for infectious diseases in animals and humans worldwide. Cattle and poultry have been implicated as major sources of *Salmonella* contaminated food products that are responsible for human salmonellosis [1, 2]. Several serovars also regularly cause disease in animals and humans by transmission from one individual to another. *Salmonella* is difficult to control in food and animal environments, since animals may be asymptomatic fecal shedders. These ‘‘carrier” animals likely play an important role in the spread of infection between herds and flocks and consequently serve as sources of food contamination and human infection [3, 4].

In industrialized countries, it is primarily the economic and social burden of these infectious diseases that is important. In the U.S.A, the annual costs of salmonellosis are estimated between 400 million and 3.5 billion U.S. dollars for the whole American economy. These values take into account medical costs and loss of productivity [5, 6]. In Europe, considering that 95% of salmonellosis cases are food-borne infections, annual costs range between 560 million and 2.8 billion euros [3]. In addition to its pathogenicity, *Salmonella* isolates that are resistant to antimicrobials have become a worldwide health issue in recent years [7].

In Africa, non-typhoidal *Salmonella* (NTS) are a major cause of severe invasive disease in adults and children. Infants and young children less than 3 years of age are the most vulnerable, and clinical associations have been reported with malaria, anaemia, malnutrition, and more recently with HIV infection [8]. HIV infection with advanced immunodeficiency is the major risk factor for invasive NTS disease in African adults. The predominant NTS serovars in the African region are *Salmonella* Typhimurium and *Salmonella* Enteritidis which are increasingly resistant to many antimicrobial agents [9, 10]. *Salmonella* Enteritidis is considered to be an important human pathogen worldwide in the past decade. The consumption of eggs or derived products has been associated with a high percentage of human *S*. Enteritidis outbreaks [11, 12]. Keddy et al. [13] reported in their study 652 invasive *Salmonella* Typhimurium from patients in a provincial hospital in South Africa between 2006 and 2007. These patients were aged 15–64 years and 93% of those tested were infected with HIV, responsible for AIDS.

In Chad, *Salmonella* strains and other important bacterial pathogens are not often isolated and identified, and the resistance of these pathogens including *Salmonella* to commonly used antibiotics is rarely assessed or not at all looked for. Furthermore, the use of antibiotics for treatment of human enteric infections is not subject to any regulation. The aims of this present study were therefore, to investigate the presence and antimicrobial resistance of *Salmonella* isolates in humans and their dissemination in N’Djamena region.

## Materials and Methods

### Sampling

N’Djamena autonomous region has 11 functional hospitals and several private clinics whose geographic locations depend on the importance of population density in urban districts. Five hospitals agreed to participate in our study (Table 1). Permit to collect samples in Hospital 4 was obtained later during the study and that’s the reason why less samples were collected. Our study was carried out during two seasons, from August 2010 to January 2011 and from September 2011 to February 2012, and involved patients, in- or out- patients suffering from diarrhea in these five hospitals (four public and one private) in urban area of N’Djamena. Only one hospital was sampled each week, ten samples being collected on a single appointed visit on the second day of the week. Each sample was collected from laboratories of these health centers in sterile pouches (AES Chemunex, Combourg, France), placed in a cool box with ice packs and immediately transported to the Laboratory of Veterinary and Zootechnical Research (LRVZ/ N’Djamena) for analysis within the same day.

**Table 1:** Prevalence of *Salmonella* strains isolated in 5 N’Djamena hospitals, Chad.

Hospitals	Number of human faeces samples (n = 420)	
	Examined	Positive (%, [95%CI])
Hosp 1	90	6 (7%, [1.5–11.8])
Hosp 2	90	4 (4%, [0.2–8.7])
Hosp 3	90	1 (1%, [0–3.3])
Hosp 4	60	11 (18%, [8.5–28.1])
Hosp 5	90	17 (19%, [10.8–27.0])
**Total**	**420**	**39 (9.3%, [6.5–12.1])**

### Bacterial isolation and characterization

Stool cultures were performed using food microbiology standard selective and enrichment culture techniques [14]. All *Salmonella*-like colonies were identified to the genus level by their biochemical characteristics using the Microgen ID-GNA gallery for enterobacteria (AES Chemunex, Combourg, France). *Salmonella* strains were serotyped according to the White-Kauffmann-Le Minor Scheme [15]. To reduce experiment time and cost linked to the classical serotyping method, Premi Test *Salmonella* System (PTS) Kit (Check-Points BV, Wageningen, The Netherlands) was also used for typing some *Salmonella* isolates [16]. Antimicrobial susceptibility was tested by disk diffusion according to the CLSI (Clinical and Laboratory Standards Institute) recommendations [17]. The 16 antibiotics tested were ampicillin (10 μg), amoxicillin+clavulanic acid (20, 10 μg), cephalotin (30 μg), cefotaxime (30 μg), ceftazidime (30 μg), sulphonamides (300 μg), cotrimoxazole (1.25/23.75 μg), gentamicin (10 μg), streptomycin (10 μg), kanamycin (30 μg), tetracycline (30 μg), chloramphenicol (30 μg), colistin (50 μg), nalidixic acid (30 μg), ofloxacin (5 μg), and enrofloxacin (5 μg) (Oxoid, Dardilly, France). Zone diameters were read by the automated Osiris scanner (Bio-Rad, Marne la Coquette, France) and interpreted with CLSI guidelines [18]. *Escherichia coli* ATCC 25922 was used as quality control. The set of these methods were carried out as previously described [19] at the French National Reference Laboratory for antimicrobial resistance. *qnrA*, *B*, *S* gene detection was performed by multiplex PCR as previously described [20].

### Ethics statement

This study was approved by the Regional Health Delegation of N’Djamena, Public Health Ministry, Chad, and by the Review Board of the N’Djamena University. All adult subjects provided informed consent, and a parent or guardian of any child participant provided informed consent on their behalf. Informed consent was oral, as the majority of the people are illiterate in Chad. Informed consent was collected by the technicians in charge of samples, and recorded in laboratory notebooks. However, one must emphasize all the data were analyzed anonymously.

## Results

A total of 420 samples of human faeces collected from 5 N’Djamena hospitals were analysed for the presence of *Salmonella* and of these, 39 (9.3% %) samples were positive for *Salmonella* (Table 1). Out of the total of 39 positive samples, 43 *Salmonella* isolates were identified, belonging to 21 different serovars. The most frequent serovar was *Salmonella* Stanleyville (n = 7), followed by *S*. Anatum (n = 4) and *S*. Kottbus (n = 3). The other serovars were under-represented. Descriptions of each *Salmonella* isolate serovar, source and resistance profile are shown in Table 2. Among the 43 *Salmonella* isolates tested for resistance to 16 different antimicrobials, only 2 (less than 5%) *S*. Enteritidis isolates were found resistant to nalidixic acid, ofloxacin and ciprofloxacin (Table 2). The resistance mechanism involved in this low level of resistance to fluoroquinolone was investigated by multiplex PCR. Both isolates were carrying a *qnrB* gene. The 2 fluoroquinolone resistant *S*. Enteritidis were isolated in the same hospital at the same date from 2 different patients and no link has been established between those 2 patients.

**Table 2:** Distribution and antimicrobial susceptibility profiles of *Salmonella* serovars isolated from 5 N’Djamena hospitals, Chad.

Hospitals Isolates per hospital	Serovars	Isolates sources by Gender/age of patients	Number of patients per serovar	Antimicrobial Resistance profiles
**Hosp5** 19 isolates of 9 serovars	*S*. Anatum	F/17ys, M/48ys, F/50ys, F/20ys^a^	4	Pan-susceptible
	*S*. Braenderup	M/20ys, F/5ys,	2	Pan-susceptible
	*S*. Stanleyville	M/34ys^b^, M/13ys, F/18ys	3	Pan-susceptible
	*S*. Altona	M/6ys, M/16ys	2	Pan-susceptible
	*S*. Farcha	M/38ys, F/20ys^a^	2	Pan-susceptible
	*S*. Hato	M/32ys, M/52ys	2	Pan-susceptible
	*S*. Havana	F/12ys	1	Pan-susceptible
	*S*. Urbana	M/59ys, M/22ys	2	Pan-susceptible
	*S*. Hull	M/34ys^b^	1	Pan-susceptible
**Hosp4** 12 isolates of 6 serovars	*S*. Stanleyville	M/32ys, F/66ys, F/32ys, F/15ys	4	Pan-susceptible
	*S*. Colindale	F/23ys, F/16ys	2	Pan-susceptible
	*S*. Idikan	F/14ys, M/1ys	2	Pan-susceptible
	*S*. Enteritidis	M/23ys, M/15ys	2	NAL, OFX, CIP
	*S*. Teshie	M/30ys^c^	1	Pan-susceptible
	*S*. Vom	M/30ys^c^	1	Pan-susceptible
**Hosp1** 6 isolates of 4 serovars	*S*. Herston	F/2ys, F/50ys	2	Pan-susceptible
	*S*. Ona	M/24ys, F/22ys	2	Pan-susceptible
	*S*. Kottbus	F/12ys	1	Pan-susceptible
	*S*. 8:i:-	F/31ys	1	Pan-susceptible
**Hosp2** 5 isolates of 3 serovars	*S*. Kottbus	M/54ys, F/14ys^d^	2	Pan-susceptible
	*S*. Amsterdam	M/45ys, F/6ys	2	Pan-susceptible
	*S*. 6, 7:a:-	F/14ys^d^	1	Pan-susceptible
**Hosp3** 1 isolates of 1 serovar	*S*. Muenchen	F/2ys	1	Pan-susceptible
**Total**43 isolates of 21 serovars	**21 serovars**	**39 patients**	**43 isolates**	**2 profiles**

ys: years; M: male; F: female;

^a, b, c, d^: patient followed by the same superscript letter = same patient; Pan-susceptible means susceptible to all antimicrobials tested; NAL: nalidixic acid, OFX: ofloxacin, CIP: ciprofloxacin

## Discussion

We identified several serovars of *Salmonella* at the end of our epidemiological investigations in the N’Djamena hospitals. These human *Salmonella* strains were isolated from a single type of stool samples in the following clinical situations: gastroenteritis, diarrhea and dysentery. In this study, 9.3% of 420 faeces samples collected from 5 hospitals in the capital city and examined by stool cultures were positive for *Salmonella* (Table 1). This prevalence rate observed highlights an alarming clinical picture knowing that all *Salmonella* serovars can, theoretically, cause a systemic infection in humans having a weak immune status [21]. This study provides a first recent description of the human *Salmonella* serovars present in the N’Djamena area.

In most African countries, non-typhoidal *Salmonella* is cited among the three most common pathogenic bacteria, responsible for bloodstream infections in adults and children. The cases of death related to diseases due to invasive non typhoidal *Salmonella* among hospitalized patients range from 4.4% to 27% in children and 22% to 47% in adults [22, 9].

From one African country to another, these prevalence data may vary depending on socio-economic and demographic situations. The study conducted by Hill et al. [23] revealed that non-typhoidal *Salmonella* represented 8.6% of bacteraemia cases, especially linked to young children in Gambian hospitals. In Senegal, relying on the data obtained on patients with HIV by Dakar University Hospital from 1996 to 2005, Seydi et al. [24] reported 62 cases (8.8%) of bacteraemia due to non-typhoidal *Salmonella*. In Malawi, 64% of newborns admitted to a pediatric hospital died after meningitis caused by non-typhoidal *Salmonella* [9]. Kariuki et al. [25] reported through their study, a total of 336 non-typhi *Salmonella* isolated from blood cultures from 1994 to 2005 in children aged 0–13 years admitted to a rural pediatric hospital in Kenya. This must be stressed that these studies focused on one clone, namely Typhimurium ST313. Nevertheless, co-factors like HIV will favour invasive Salmonella infection whatever the serovar.

Indeed, far from being exhaustive, these data show the importance of human salmonellosis in different African countries. It must be emphasized that the prevalence rate (9.3%) revealed by this study does not take into account specifically the age group or sex, and the immune status of respondents. It rather highlights a global situation of non-typhoidal *Salmonella* gastroenteritis in patients of all ages and both genders who consulted the five hospitals of N’Djamena involved in our study (Table 2). Before this study in these five N’Djamena hospitals, the medical diagnosis of gastroenteritis and other human food-borne infections was never oriented by clinicians, to a suspicion of possible salmonellosis. Furthermore, to discuss *Salmonella* prevalence in humans, it is necessary to look back to the 1960s and ‘70s to gather information on *Salmonella* serovars in the N’Djamena area [26, 27]. Results from this study provide therefore, relevant information to clinicians about diagnosis and medical care of human gastroenteritis and other severe infections that can be caused by *Salmonella* serovars: one out of ten patients consulting on gastroenteritis purposes are infected by *Salmonella*.

In the present study, the analysis of stool samples from 5 hospitals in N’Djamena, revealed many isolates of *Salmonella*, which were particularly heterogeneous. All strains were isolated from patients with episodes of digestive pain and diarrhea. We identified a total of 43 *Salmonella* isolates belonging to 21 different serovars. The diversity of serovars observed in humans in this study reflects the singularity of this result, unlike results published by Galanis et al. [3] and EFSA [4] concerning industrialized countries and many African countries where the distribution of *Salmonella* serovars seems quite homogeneous. However, one of the studies conducted in N’Djamena in the 60s and 70s already pointed out such a variety of isolated serovars, leading to the description of 10 previously unidentified serovars [26].

The different serovars identified are characterized by their specific distribution linked to the geographical location of each hospital. Only serovars Kottbus and Stanleyville were found in two different hospitals located in two urban areas of N’Djamena quite far from each other. Most of these serovars were already recovered by Le Minor et al. [26] and Guard et al. [27] in stool samples collected, at least fourty years ago, at the main hospital of Fort-Lamy region, renamed meanwhile N’Djamena.

In Africa, despite monitoring systems, non-typhoidal *Salmonella* serovars remain the leading causes of infant bacteraemia and are responsible for high rates of morbidity and mortality, particularly among young children and elderly or immunocompromised people, when treatment with appropriate antibiotics is not available [25, 28, 29]. Based on the work of Vandenberg et al. [10], out of 59% of invasive non-typhi *Salmonella* isolated from blood cultures of children in a rural district hospital of the Democratic Republic of Congo, where the malaria disease is endemic, the most represented serovars were *S*. Typhimurium (61%) and *S*. Enteritidis (22%). The mortality rate associated with these serovars was of 23% and most of the isolates were resistant to multiple antimicrobial (92%).

Out of the total of 43 *Salmonella* serovars isolated from humans in this study, only two *S*. Enteritidis were resistant to nalidixic acid, ofloxacin and ciprofloxacin. Even if no link has been established at the hospital between those 2 patients, Hospital 4 gathers patients from 6 wards of N’Djamena, shopping exclusively on the same single market and offering a unique big high school; we cannot assess that those 2 men have nothing in common. The other *Salmonella* isolates were pan-susceptible to the 16 antimicrobials tested. The emergence of quinolone resistance in the most common *Salmonella* serovar worldwide is a serious public health concern. Resistance to nalidixic acid has been associated with reduced efficacy of fluoroquinolones such as ciprofloxacin. Several studies conducted in many countries worldwide have demonstrated that *Salmonella* Enteritidis encountered in animal and human infections have showed various resistances to antimicrobial molecules, particularly to quinolones, including fluoroquinolones [30]. This finding is of concern because quinolone and fluoroquinolone molecules are first-line drugs for ambulatory treatment of human invasive salmonellosis. All these data highlight the real burden of non-typhoidal *Salmonella* infections in African countries and the associated risk factors. It therefore appears necessary to improve the current knowledge on food and environmental risk factors linked to the mechanisms of *Salmonella* contaminations and infections. The results shown by this study in hospitals of N’Djamena region are very significant and complete this severe epidemiological situation of *Salmonella* infections in the African context. Also, we point out that the comparison with avian *Salmonella* isolated at the same period using some molecular methods to assess the possible contribution of avian *Salmonella* isolates to human salmonellosis is under way.

This study provides data concerning prevalence rate (9.3%) and resistance to antimicrobials of *Salmonella* isolates among human in N’Djamena, Chad. In the course of this study, we revealed the great diversity among the *Salmonella* serovars isolated from human samples in the different health centers investigated and have shown a relatively low proportion of these isolates resistant to antimicrobial agents in the N’Djamena area. Currently, the real burden of human diseases due to *Salmonella* serovars in Chad remains unknown. A comprehensive epidemiological investigation of invasive non-typhoidal *Salmonella* and diarrhea caused by non-typhoidal *Salmonella* is needed. A clearer understanding of the incidence, complications, and case-fatality rates related to non-typhoidal *Salmonella* serovars at the population level would also be important in deciding how to improve health care management in N’Djamena, Chad.
